# Supplementary material for: Ionizing Radiation Sensing with Functionalized and Copper-Coated SWCNT/PMMA Thin Film Nanocomposites
Source: Nanomaterials (Basel). 2023 Sep 27;13(19):2653. doi: 10.3390/nano13192653 (PMC10574655; doi:10.3390/nano13192653)
Supplement: Supplementary file 1 [file nanomaterials-13-02653-s001.zip › nanomaterials-2623890-supplementary.pdf]

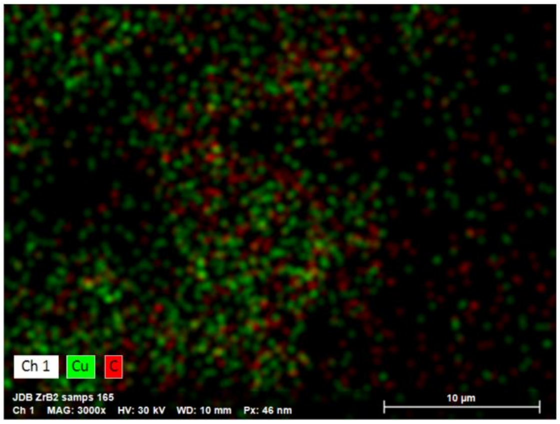

Figure S1. Shows the EDS characterization of thin copper-coated SWCNT/PMMA thin-film composite. This confirms the presence of functional group with Cu in the copper-coated SWCNT/PMMA composite.

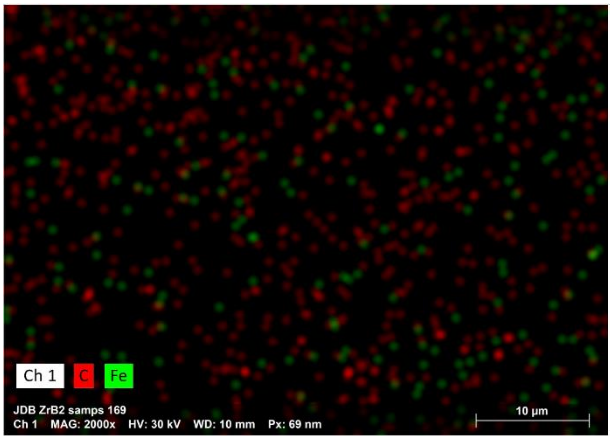

Figure S2. Shows the EDS characterization of the ferrocene-doped-SWCNT/PMMA. This confirms that ferrocene is present in the ferrocene doped-SWCNT/PMMA composite.
